# antiSMASH 7.0: new and improved predictions for detection, regulation, chemical structures and visualisation

**DOI:** 10.1093/nar/gkad344

**Published:** 2023-05-04

**Authors:** Kai Blin, Simon Shaw, Hannah E Augustijn, Zachary L Reitz, Friederike Biermann, Mohammad Alanjary, Artem Fetter, Barbara R Terlouw, William W Metcalf, Eric J N Helfrich, Gilles P van Wezel, Marnix H Medema, Tilmann Weber

**Affiliations:** The Novo Nordisk Foundation Center for Biosustainability, Technical University of Denmark, Kgs.Lyngby, Denmark; The Novo Nordisk Foundation Center for Biosustainability, Technical University of Denmark, Kgs.Lyngby, Denmark; Molecular Biotechnology, Institute of Biology, Leiden University, Leiden, The Netherlands; Bioinformatics Group, Wageningen University, Wageningen, The Netherlands; Bioinformatics Group, Wageningen University, Wageningen, The Netherlands; Bioinformatics Group, Wageningen University, Wageningen, The Netherlands; Institute of Molecular Bio Science, Goethe-University Frankfurt, Frankfurt am Main, Germany; LOEWE Center for Translational Biodiversity Genomics. Frankfurt am Main, Germany; Bioinformatics Group, Wageningen University, Wageningen, The Netherlands; Bioinformatics Group, Wageningen University, Wageningen, The Netherlands; Institute of Technical Chemistry, Leibniz University Hannover, Hannover, Germany; Bioinformatics Group, Wageningen University, Wageningen, The Netherlands; Department of Microbiology, University of Illinois Urbana–Champaign, Urbana, IL, USA; Institute for Genomic Biology, University of Illinois Urbana–Champaign, Urbana, IL, USA; Institute of Molecular Bio Science, Goethe-University Frankfurt, Frankfurt am Main, Germany; LOEWE Center for Translational Biodiversity Genomics. Frankfurt am Main, Germany; Molecular Biotechnology, Institute of Biology, Leiden University, Leiden, The Netherlands; Bioinformatics Group, Wageningen University, Wageningen, The Netherlands; The Novo Nordisk Foundation Center for Biosustainability, Technical University of Denmark, Kgs.Lyngby, Denmark

## Abstract

Microorganisms produce small bioactive compounds as part of their secondary or specialised metabolism. Often, such metabolites have antimicrobial, anticancer, antifungal, antiviral or other bio-activities and thus play an important role for applications in medicine and agriculture. In the past decade, genome mining has become a widely-used method to explore, access, and analyse the available biodiversity of these compounds. Since 2011, the ‘antibiotics and secondary metabolite analysis shell—antiSMASH’ (https://antismash.secondarymetabolites.org/) has supported researchers in their microbial genome mining tasks, both as a free to use web server and as a standalone tool under an OSI-approved open source licence. It is currently the most widely used tool for detecting and characterising biosynthetic gene clusters (BGCs) in archaea, bacteria, and fungi. Here, we present the updated version 7 of antiSMASH. antiSMASH 7 increases the number of supported cluster types from 71 to 81, as well as containing improvements in the areas of chemical structure prediction, enzymatic assembly-line visualisation and gene cluster regulation.

## Graphical Abstract

**Graphical Abstract:**
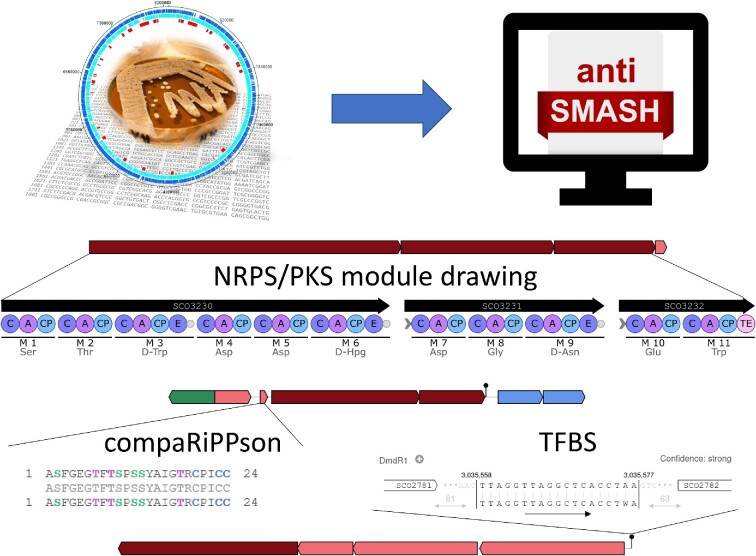
antiSMASH 7 increases the number of supported cluster types from 71 to 81, as well as containing improvements in the areas of chemical structure prediction, enzymatic assembly-line visualisation and gene cluster regulation.

## INTRODUCTION

Small bioactive compounds produced by microorganisms form the basis of many drugs (1) and crop protection agents (2). Traditionally, new compounds were discovered using a ‘find and grind’ workflow of extracting from natural sources, chemically isolating, purifying, and then testing compounds. This approach is now routinely complemented by sequencing and subsequent mining of genome and metagenome data to identify natural product biosynthetic pathways (3). Software tools for ‘genome mining’, i.e. searching genomes for secondary/specialised metabolite (SM) biosynthetic gene clusters (BGCs), have existed for over a decade (4–7).

Since its 2011 release, antiSMASH (8–13) has established itself as the most widely used tool for mining microbial genomes for SM BGCs. Around antiSMASH, an ecosystem of independent tools that incorporate or utilise antiSMASH results has developed, such as the antibiotics resistance target seeker (ARTS 2) (14), the mass-spectrometry-guided peptide mining tool Pep2Path (15), the sgRNA design tool CRISPY-web 2 (16), the BGC networking and clustering platform BiG-SCAPE (17), and the related big data BGC clustering tool BiG-SLiCE (18). In turn, antiSMASH can also incorporate and display BGC predictions from other tools such as DeepBGC (19) by using the sideloading mechanism introduced in antiSMASH 6 (13). antiSMASH BGC predictions are included in many genomic and BGC-oriented databases, like the Joint Genome Institute's Integrated Microbial Genomes database with its Atlas of Biosynthetic gene Clusters IMG-ABC (20), the MicroScope platform for microbial genome annotation and analysis (21), the MIBiG database of manually curated BGCs (22), the BGC family database BiG-FAM (23) and the antiSMASH database (24).

antiSMASH uses a rule-based approach to identify many different types of biosynthetic pathways involved in SM production. More in-depth analyses are performed for BGCs encoding non-ribosomal peptide synthetases (NRPSs), type I and type II polyketide synthases (PKSs), and the ribosomally synthesised and post-translationally modified peptide (RiPP) classes of lanthipeptides, lasso peptides, sactipeptides, and thiopeptides. For these, cluster-specific analyses can provide more information about the biosynthetic steps performed and thus also provide more detailed predictions on the compound(s) produced.

Here, we present version 7 of antiSMASH. It improves upon and further extends previous versions by adding and updating BGC detection rules, enhancing regulatory function detection by predicting transcription factor binding sites represented in the LogoMotif database (https://logomotif.bioinformatics.nl/), and adding new visualisations for NRPS and PKS clusters, PFAM (25) and TIGRFAM (26) domain hits, as well as tables listing all genes in a region with dynamic search and filter functions.

## NEW FEATURES AND UPDATES

### New cluster types and dynamic detection profiles

antiSMASH uses manually curated and validated ‘rules’ that define which core biosynthetic functions need to exist in a genomic region in order to constitute a BGC. To identify these biosynthetic functions, antiSMASH uses profile hidden Markov models (pHMMs) from PFAM (25), TIGRFAMs (26), SMART (27), BAGEL (28), Yadav *et al.* (29) and custom models. antiSMASH 6 contained rules for 71 BGC types (13). In antiSMASH 7, this number increases to 81, adding support for 2-deoxy-streptamine aminoglycosides, aminopolycarboxylic acid metallophores, arginine-containing cyclo-dipeptides (RCDPs), crocagins, methanobactins, mycosporines, NRP-metallophores (30), opine-like metallophores, and fungal-RiPP-likes. NRP metallophore BGCs were previously detected by the general NRPS detection rules, but are now recognised specifically on the basis of genes encoding the biosynthesis of functional groups involved in metal chelation (30). The phosphonates rule was updated, with the old rule retained under the name of ‘phosphonate-likes’. In addition to an improved phosphoenolpyruvate (PEP) mutase detection model, supporting models (Supplementary Tables S1-S2) are leveraged to reduce false positives and improve delineation of cluster boundaries (Supplementary Figure S1).

Because not all features of a BGC can be captured with pHMMs, antiSMASH 7 adds the option of creating dynamic profiles that are defined by Python code instead. This is currently being used to detect cyanobactin precursors based on the M.KKN[IL].P….PV.R motif as described in (31), a conserved sequence motif too small to be picked up in a pHMM reliably.

### NRPS & PKS improvements

To improve PKS annotations in fungal gene clusters, we have added detection profiles for carnitine-AT (cAT), product template (PT) and thiocysteine/beta-eliminating lyase (SH) domains. The ketosynthase (KS) domains of bacterial trans-acyltransferase polyketide synthases (trans-AT PKSs) are now also annotated using the subtype-specific pHMMs of transATor (32). PKS KS domains and NRPS condensation (C) domains can be submitted to the recently released version 2 of the Natural Product Domain Seeker (NapDoS2) (33) for phylogenetic analysis. The recent MIBiG 3 release (22) adds substrate specificities for over 2000 NRPS adenylation (A) and related domains. To allow our users to benefit from the additional information, we have replaced the NRPSPredictor2 A domain substrate prediction tool (34) we have been shipping since 2011 with the new ‘NRPyS’ library (https://github.com/kblin/nrpys/) that allowed us to update the Stachelhaus code (35) lookup table from previously 554 to now 2319 entries. As the 10 amino acid (AA) code used by Stachelhaus does not always resolve to a single substrate prediction in the new data set, most likely due to substrate promiscuity of the A domain involved, NRPyS reports all equal quality 10 AA code hits, ranked by the highest match to the 34 AAs predicted to be in an 8 Å radius around the A domain active site following the description of Rausch et al. used in NRPSPredictor 1 (36). To act as a full drop-in replacement, NRPyS still runs the original support vector machine (SVM) models from NRPSPredictor 2.

### RiPP precursor comparisons

**Figure 1.**
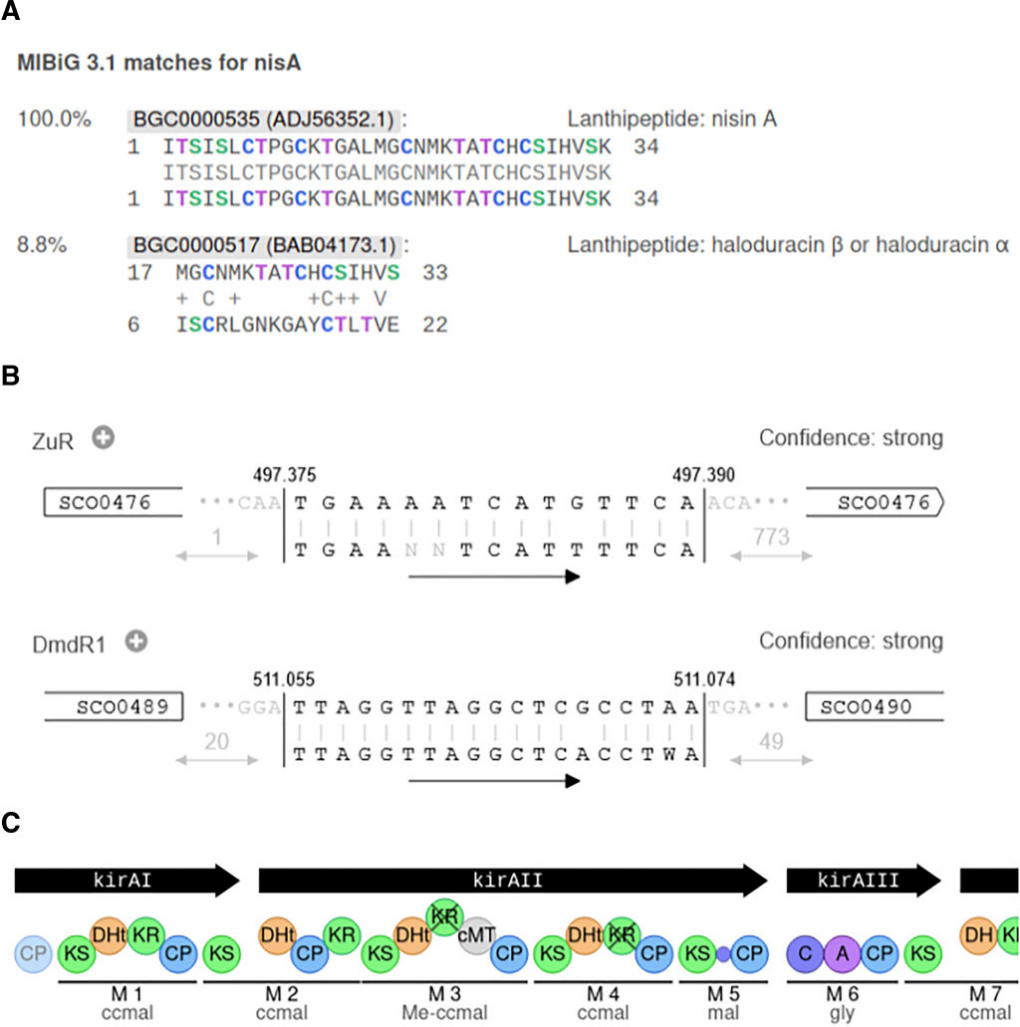
Examples of new antiSMASH visualisations. (**A**) shows the CompaRiPPSon MIBiG matches for the lanthipeptide class I nisin A input sequence with a 100% match for the self hit, and a much lower (8.8%) match with another lanthipeptide. (**B**) shows two high confidence TFBS-finder hits on *Streptomyces coelicolor* A3(2). The first hit, a putative ZurR binding site, is located right at the start of gene SCO0476, with the last two bases of the ATG start codon being the first two bases of the binding site. The DmdR1 hit is located between and upstream of both SCO0489 and SCO0490. (**C**) shows the first modules of the *Streptomyces colinus* Tü 365 hybrid trans-AT PKS/NRPS kirromycin gene cluster (MIBiG ID: BGC0001070).

To help users with evaluating the novelty of RiPP precursor peptides, we have developed the CompaRiPPson analysis that compares the (predicted) core peptides of identified RiPP precursors to RiPP precursors in the antiSMASH-DB (24) and MIBiG 3.1 (22) databases. Hits for these databases are presented separately, with the antiSMASH-DB hits containing a much larger dataset of 10583 predicted precursors in version 3.0 versus 28 experimentally verified and annotated precursors from MIBiG. Precursor hits are labelled by precursor gene locus tag for the antiSMASH-DB and labelled by compound name for MIBiG. Ordered by sequence identity, database hits with identical precursor sequences are grouped together. Query and hits are displayed with alignments (Figure 1A).

### Transcription factor binding site predictions

The LogoMotif database (https://logomotif.bioinformatics.nl/) contains a curated collection of experimentally validated transcription factor binding site (TFBS) profiles and corresponding position weight matrices (PWMs), focusing on Actinobacteria. The antiSMASH TFBS-finder module uses these PWMs to annotate putative TFBSs. Depending on the hit score, TFBS-finder displays a confidence level of strong, medium, or weak, respectively. Binding sites are displayed in their genomic context, indicating the orientations and distances to surrounding genes (Figure 1B). All hits link to the LogoMotif website for more in-depth information about specific profiles.

### Gene table

Every region now lists all contained gene features in a filterable, interactive table. Genes can be filtered by entering a search term in the search box (plain text and regex are both supported). Genes that match the filter will be shown in the region view and, if enabled, the view will automatically zoom to the selection. Information used for filtering currently includes the name of the gene, its biosynthetic type, and gene function annotations (e.g. smCOG hits).

### Updated visualisations and other optimisations

A new visualisation for NRPS and PKS clusters draws enzymatic domains and modules in the predicted assembly order in conventional publication style, which allows researchers to use the antiSMASH vector graphics as a starting point for their publication-quality figures (Figure 1C). PFAM (25) and TIGRFAMs (26) domain hits in a region are now shown in a similar fashion to the existing NRPS/PKS domain visualisations.

Following the MIBiG 3.1 release (22), the KnownClusterBlast and ClusterCompare databases were updated.

## CONCLUSIONS & FUTURE PERSPECTIVES

Genome mining for natural product BGCs with tools like antiSMASH forms a cornerstone of modern natural product discovery workflows. With the additions and updates presented in this manuscript, antiSMASH is being continuously updated to remain the go-to solution for microbial natural product genome mining. The Open Source antiSMASH software continues to contribute to the thriving ecosystem of computational tools in the natural products field. In addition to providing microbial natural product predictions directly, antiSMASH also serves as the technology platform for other tools, such as the plant natural products prediction tool plantiSMASH (37), the primary metabolism gene cluster prediction tool gutSMASH (38,39), and other tools currently in development. In future updates, we will continue our work on improving compound structure and subcomponent predictions, adding additional TFBS profiles for different taxa (e.g. fungal profiles from JASPAR (40)), as well as integrating with other tools in the ecosystem. We have also started providing a website to try out potential future antiSMASH features at https://experimentalsmash.secondarymetabolites.org/.

## DATA AVAILABILITY

The bacterial and fungal versions of antiSMASH 7 can be freely accessed at https://antismash.secondarymetabolites.org and https://fungismash.secondarymetabolites.org, respectively.

The antiSMASH documentation is available at https://docs.antismash.secondarymetabolites.org/.

The antiSMASH source code is licensed under the GNU Affero General Public License (AGPL) v3.0. antiSMASH is also available via Docker. See the documentation website for details on how to download and install antiSMASH.

## Supplementary Material

gkad344_Supplemental_File
