# Supplementary material for: antiSMASH 7.0: new and improved predictions for detection, regulation, chemical structures and visualisation
Source: Nucleic Acids Res. 2023 May 4;51(W1):W46–50. doi: 10.1093/nar/gkad344 (PMC10320115; doi:10.1093/nar/gkad344)
Supplement: gkad344_Supplemental_File [file gkad344_supplemental_file.pdf]

Supplemental Figures and Tables

Figure S1: Demonstration of the improved phosphonate BGC boundary conditions in comparison to the old model.

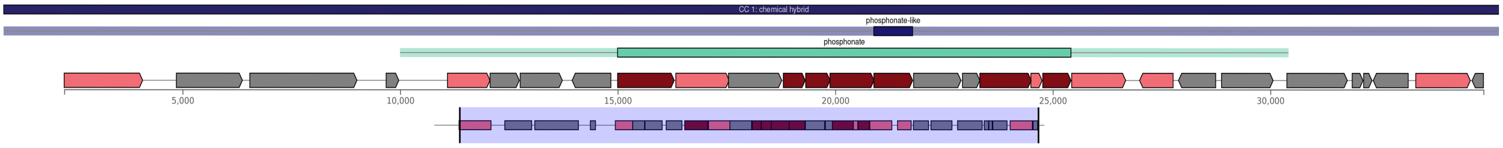

(a) Rhizocticin A BGC (MiBiG: BGC0000926), a known phosphonate BGC. Dark-blue (phosphonate-like): detection as performed by the old phosphonate model. The detection includes only the pepM gene (dark blue block) and extends the detection area far beyond the most relevant secondary core genes (dark red blocks). Turquoise: detection as performed by the new model, the detection now includes the pepM gene and all relevant secondary core genes (dark red), but does not extend as far from the first and last core gene in the cluster. This much smaller but more relevant genetic context of the phosphonate BGC is given out upon the BGC detection.

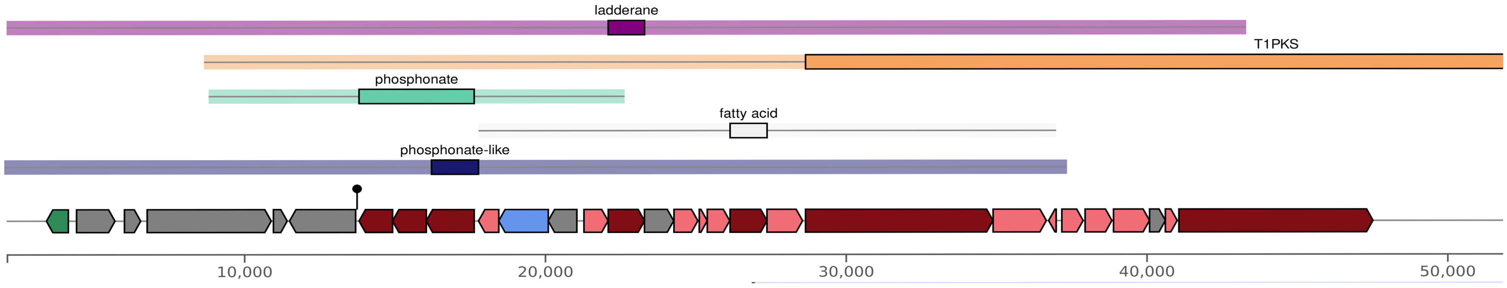

(b) Selected result of an antiSMASH screen on Assembly accession number GCF_000966965.1. Example of a genetic region likely containing multiple adjacently located but different BGCs. The old model (phosphonate-like, dark blue) includes regions of neighboring clusters into the detected phosphonate BGC and thus introduces irrelevant genetic context into the cluster. The new model (phosphonate, turquoise) limits itself to only the primary and secondary core genes and adds less genetic neighborhood to the detected BGC. The new model overlaps much less with the neighboring example BGCs of the ladderane (purple), T1PKS (yellow) and fatty acid (white) types.

Table S1: Summary of supplemental models representing enzymes known in phosphonate biosynthesis reported in doi: 10.1099/mgen.0.000781.

Name	Description	PFAM
2-Hacid_dh_C	D-isomer specific 2-hydroxyacid dehydrogenase, NAD binding domain	PF02826
Aldedh	Aldehyde dehydrogenase family	PF00171
Aminotran_1 2	Aminotransferase class I and II	PF00155
Aminotran_5	Aminotransferase class-V	PF00266
AurF	P-aminobenzoate N-oxygenase AurF	PF11583
CTP_transf_like	Cytidylyltransferase-like	PF01467
DUF4992	Domain of unknown function	PF16383
Fe-ADH	Iron-containing alcohol dehydrogenase	PF00465
HMGL-like	HMGL-like	PF00682
Metallophos_2	Calcineurin-like phosphoesterase superfamily domain	PF12850
NTP_transf_3	MobA-like NTP transferase domain	PF12804
NTP_transf_5	Uncharacterised nucleotidyltransferase	PF14907
PALP	Pyridoxal-phosphate dependent enzyme	PF00291
PEP_mutase	Phosphoenolpyruvate phosphomutase	PF13714
TPP_enzyme_C	Thiamine pyrophosphate enzyme, C-terminal TPP binding domain	PF02775
TPP_enzyme_N	Thiamine pyrophosphate enzyme, N-terminal TPP binding domain	PF02776

Table S2: Detection models used with associated bit score cutoffs. Improved PepM model composed of sequences with characteristic EDK(X..)NS motif. Closely related enzymes in the isocitrate lyase family (phosphonopyruvate hydrolase and methylisocitrate lyase) are used for negative filtering to reduce known false positives.

Description	Score cutoff	Model
Phosphonate biosynthesis PEP mutase	150	phosphonates.hmm
Phosphonopyruvate hydrolase	400	phosphonatesHydrolase.hmm
Methylisocitrate Lyase	250	phosphonatesMeIsocitrateLyase.hmm
